# ACDF vs TDR for patients with cervical spondylosis – an 8 year follow up study

**DOI:** 10.1186/s12893-017-0316-9

**Published:** 2017-11-28

**Authors:** Bolong Zheng, Dingjun Hao, Hua Guo, Baorong He

**Affiliations:** Department of Spine Surgery, Honghui Hospital, Xi’an Jiaotong University, NO555,East Friendship Rd, Xi’an, Shanxi 86710054 China

## Abstract

**Background:**

ACDF has been considered as the gold standard in the treatment of single level cervical disk protrusion. However, it may cause adjacent level degeneration due to regional biomechanical changes. TDR has been applied with satisfactory results for over a decade, but there is no consensus if TDR is safer and more efficient than ACDF. The current study was carried out to compare the efficiency and safety of TDR and ACDF in the treatment of patients with single level cervical disk protrusion.

**Methods:**

One hundred forty-five consecutive patients who underwent either TDR or ACDF in our center were included in the current study. Time of surgery, intraoperative blood loss, VAS arm and neck pain scores, ROM, ODI, SF36 and Patient satisfaction were compared before the surgery, after the surgery, and during follow up 1, 3, 5, 8 years after the surgery.

**Results:**

The time of surgery was 64.6 ± 20.7 min in the ACDF group and 69.4 ± 19.3 min in the TDR group; intraoperative hemorrhage was 67.2 ± 14.3 ml in ACDF group and 70.7 ± 18.6 ml in TDR group. There were no significant differences between two groups concerning time of surgery and intraoperative blood loss. No differences were found concerning patient satisfaction between the two groups during the follow up (*P* > 0.05). Significant differences were found between the groups concerning VAS arm and neck pain scores, ROM, ODI and SF36 after the surgery and during the 8 year follow up.

**Conclusion:**

TDR may be a more effective approach than ACDF for treating patients with single level cervical disk protrusion.

**Keywords:**

Cervical disk herniation, ACDF, TDR, Retrospective study

## Background

Cervical spondylosis is one of the most common diseases among the elderly population. The typical symptoms may include pain, numbness and weakness of the shoulders and arms, some patients may also suffer from weakness of the legs and trohow, and trouble in keeping balance while walking [1–3].

Surgical intervention is necessary with patients who failed to achieve satisfactory alleviation with non-surgical treatment. For the treatment of cervical spondylosis, anterior cervical discectomy and fusion (ACDF) has been used for decades, and have been proven to be a safe and effective method, however, it has complications such as accelerated adjacent level degeneration and restricted cervical mobility. There are several studies reporting that TDR (total disk replacement) has the similar or even better therapeutic effect than ACDF. However, there is consensus on the better approach for cervical spondylosis [4–6]. In the current study, we have retrospectively reviewed the clinical materials of patients who received either ACDF or TDR for the treatment of single level cervical disk herniation, and had follow up visits for 8 years.

## Methods

A retrospective review of patients who received either ACDF or TDR in our department was carried out with approval of the ethical committee of Honghui Hospital, Xi’an Jiaotong University. All the procedures followed the guidelines of Helsinki Declaration.

### Patient inclusion

Among patients who received either ACDF or TDR in our department from January 2006 to January 2009 and followed for as long as 8 years were included in the current retrospective study. Patients with severe osteophytes and the ossification of posterior longitudinal ligament were excluded because ACDF was preferred for those patients over TDR due to the likelihood of spontaneous fusion. At the final analysis, there were 64 patients who received TDR and 81 patients who received ACDF. Clinical materials of those patients were retrospectively reviewed and analyzed at the end of the follow up.

### Surgical intervention

Patients with single level cervical disk herniation and failed to achieve satisfactory recovery after conservative treatment were treated with either ACDF or TDR after careful evaluation of overall physical and mental status. The choice between ACDF and TDR was made by the patient after being provided detailed information about both surgical procedures by the surgeon.

All the patients gave a signed consent to accept surgical treatment before the surgery. Both ACDF and TDR were carried out with patient in supine position. The cervical disk was exposed by Smith- Robinson approach [7].

**Fig. 1:**
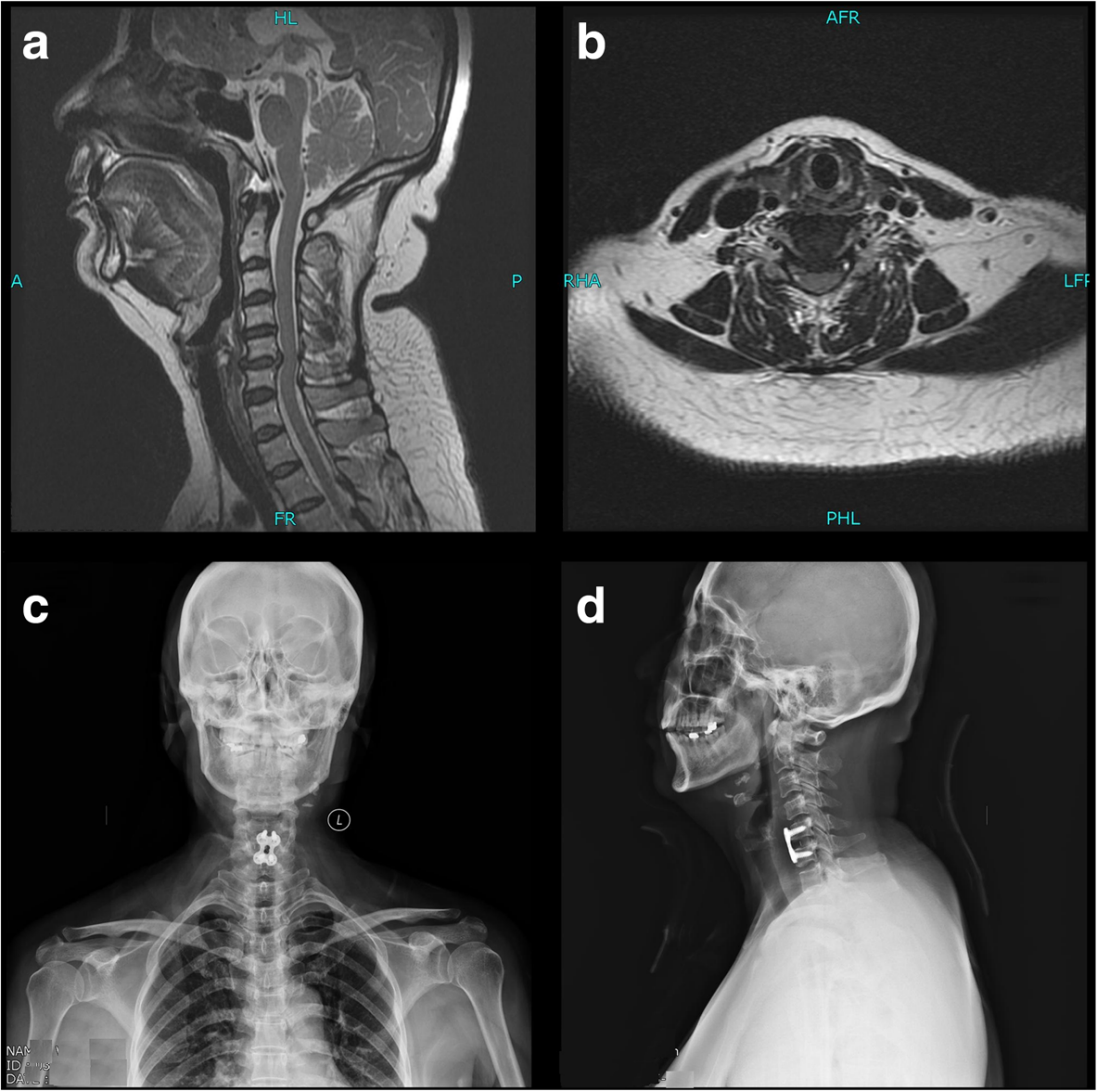
A patient with the indications of TDR: preoperative (**a**, **b**) and postoperative (**c**, **d**) radiological manifestations

In ACDF group, After exposure of the appropriate vertebral level was confirmed, the osteophytes were removed by a rougher, nucleus pulposus was dissected by nucews pulposus forceps, and the cartilaginous end plates were removed by a high speed burr. Antero-posterior fluoroscopy was applied to determine the location of the implant. The cervical vertebrae were fused by interbody cages and allogeneic bone, and fixed with a 4 screw titanium plate (Johnson and Johnson Professional Inc., Raynham, MA, USA) (Fig. 1).

**Fig. 2:**
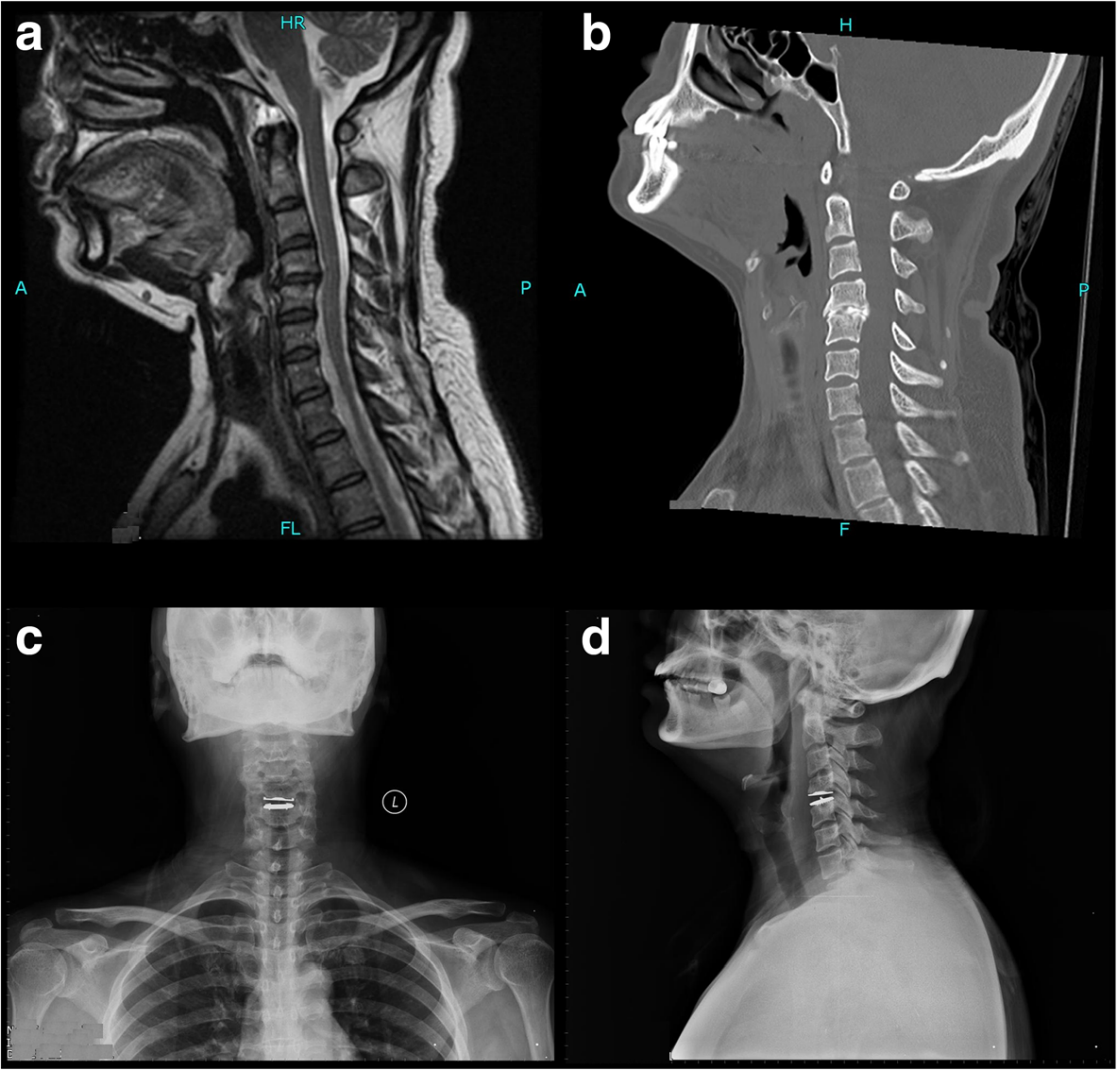
A patient with the indications of ACDF: preoperative (**a**, **b**) and postoperative (**c**, **d**) radiological manifestations

In the TDR group, anterior discectomy was performed and the location of the prosthesis was confirmed by the above mentioned procedure. Appropriate-sized prosthetic endplates (BryanTM disc (Medtronic, Minneapolis, Minnesota, USA) were inserted to an adequate depth under lateral fluoroscopic guidance (Fig. 2).

Oral pain medications were applied to ease postoperative pain for all the patients. Soft cervical collar was used to protect the patients up to a month after the surgery. Patients were encouraged to attend low intensity daily activities after the removal of the collar, and were told to engage in normal activities including sports 3 months after the surgery.

### Parameters

Time of surgery, intraoperative blood loss, visual analog scale (VAS) [8], range of motion (ROM) [9], Oswestry disability index (ODI) [10] and short form 36 item scores health survey scores (SF36) [11] were recorded before and after the surgery and 1, 3, 5, 8 years after the surgery, the parameters were recorded following the instructions in the current literature [8–11]. At each follow up interview, patients were asked if they were satisfied with the results of the surgery, and the results were recorded as patient satisfaction.

### Statistical analysis

SPSS 22.0 (IBM SPSS Statistics for Windows, Version 22.0, Armonk, NY) statistical software was used for all the statistical analysis. Independent sample t-tests were used to compare the parameters between two groups at each time point. The difference was considered significant when *P* < 0.05.

## Results

### General patient information

One hundred ninty patients received surgical treatment for single level cervical disk diseases in our department from January 2006 to January 2009. Among those patients, 145 patients were followed for as long as 8 years after the surgery, and the clinical materials of those patients were analyzed in the current study. There was no significant difference concerning the male/female ratio and the age of patients (Table 1). No significant difference was found concerning the operation time and intraoperative hemorrhage between the two groups (*P* > 0.05) (Table 1).

**Table 1:** The demographic characteristics of patients

		ACDF	TDR	P
Female/Male		32/49	25/39	0.55
Age		46.5 ± 7.6	47.2 ± 8.0	0.45
Level of surgery	C3/4	3	4	
	C4/5	9	9	
	C5/6	35	41	
	C6/7	12	20	
	C7/T1	5	7	
Operation time (min)		64.6 ± 20.7	69.4 ± 19.3	0.35
Intraoperative hemorrhage (ml)		67.2 ± 14.3	70.7 ± 18.6	0.46

### Treatment results

After the surgery, surgical incisions were healed without complications in both groups. All patients had pain relief. No graft fracture, sliding or resorption was observed. Seven patients in ACDF group and 5 patients in TDR group experienced temporary hoarseness. Thirteen patients in ACDF group and 8 patients in TDR group reported dysphagia, which disappeared within 2 weeks after surgery. Adjacent level degeneration was observed in 10 patients in the ACDF group and in 2 patients in TDR group.

VAS neck and arm pain scores were decreased significantly after the surgery in both groups. Pain alleviation was more significant in the TDR group than the ACDF group after the surgery and during follow up (*P* < 0.05) (Table 2).

**Table 2:** VAS neck and arm pain scores before, after the surgery and at different time points of follow up

		ACDF	TDR	*P*
VAS Neck pain	Preoperatively	6.6 ± 1.4	6.7 ± 1.6	0.40
	Before discharge	3.5 ± 2.2	2.4 ± 1.3	<0.01
	1	2.4 ± 1.2	1.1 ± 0.6	<0.01
	3	2.0 ± 1.1	0.8 ± 0.4	<0.01
	5	2.0 ± 1.2	1.0 ± 0.3	<0.01
	8	1.9 ± 1.1	1.0 ± 0.4	<0.01
VAS Arm pain	Preoperatively	6.8 ± 1.2	6.7 ± 1.4	0.36
	Before discharge	4.1 ± 1.1	2.1 ± 0.9	<0.01
	1	2.0 ± 0.9	1.1 ± 0.5	<0.01
	3	1.6 ± 0.9	0.8 ± 0.4	<0.01
	5	1.4 ± 0.9	0.8 ± 0.3	<0.01
	8	1.6 ± 0.8	0.7 ± 0.4	<0.01

ODI scores were significantly decreased after the surgery in both groups. The extent of improvement is more significant in TDR group than ACDF group after the surgery and during follow up (P < 0.05, Table 3).

**Table 3:** Changes in ODI scores before, after the surgery and during follow up visits

		ACDF	TDR	*P*
ODI	Preoperatively	41.3 ± 9.6	40.1 ± 11.2	0.28
	Before discharge	25.6 ± 4.9	15.3 ± 4.5	<0.01
	1	12.2 ± 3.6	6.4 ± 2.2	<0.01
	3	9.2 ± 2.2	5.6 ± 2.3	<0.01
	5	9.3 ± 3.2	5.1 ± 2.8	<0.01
	8	9.0 ± 2.5	4.2 ± 2.1	<0.01

All the patients were immobilized before discharge. During the follow up, range of motion was more significant in TDR group than ACDF group after the surgery and during follow up (*P* < 0.01, Table 4).

**Table 4:** Changes in ROM before, after the surgery and at different points of follow up

		ACDF	TDR	*P*
ROM	Preoperatively	20.5 ± 9.6	20.9 ± 7.2	0.40
	Before discharge	0	0	1.0
	1	12.7 ± 3.6	19.1 ± 3.3	<0.01
	3	8.2 ± 1.5	14.6 ± 3.0	<0.01
	5	5.7 ± 2.6	14.5 ± 4.3	<0.01
	8	2.3 ± 3.3	12.3 ± 3.2	<0.01

SF36-PCS and SF36-MCS were increased after the surgical treatment, and the increase was more significant in TDR group than the ACDF group (Table 5).

**Table 5:** Changes in SF36 score before, after the surgery and at different time points of follow up

		ACDF	TDR	*P*
SF36-PCS	Preoperatively	25.4 ± 7.4	26.3 ± 9.0	0.40
	Before discharge	36.0 ± 6.6	38.4 ± 5.6	0.18
	1	47.5 ± 7.0	56.3 ± 5.5	<0.01
	3	51.3 ± 6.1	61.6 ± 5.1	<0.01
	5	51.5 ± 6.7	65.4 ± 6.0	<0.01
	8	57.2 ± 6.3	72.6 ± 6.9	<0.01
SF36-MCS	Preoperatively	28.5 ± 6.2	29.1 ± 7.5	0.61
	Before discharge	35.7 ± 5.1	42.1 ± 5.2	0.04
	1	46.8 ± 6.3	55.1 ± 6.3	<0.01
	3	48.6 ± 5.5	58.3 ± 5.7	<0.01
	5	56.4 ± 5.4	65.7 ± 5.5	<0.01
	8	54.3 ± 7.6	74.3 ± 7.3	<0.01

More percentage of patients was satisfied with the treatment results after the surgery and during follow up, but the difference was not significant (Table 6).

**Table 6:** Changes in patient satisfaction before, after the surgery and at different points of follow up

		ACDF	TDR	*P*
Patient satisfaction	Before discharge	58(90.6%)	76(93.8%)	0.70
	1	53(82.8%)	73(90.1%)	0.78
	3	51(79.7%)	70(86.4%)	0.81
	5	50(78.1%)	67(82.7%)	0.97
	8	48(75.0%)	69(85.2%)	0.50

## Discussion

In the last century, ACDF was applied for the treatment of cervical spondylosis with satisfactory results in many patients. It is now considered the gold standard for the treatment of degenerative cervical diseases. Previous studies have reported that patients can achieve significant neurologic recovery and alleviation of pain after ACDF [12–14]. However, fusion of two vertebral bodies inevitably leads to increased stress on the intervertebral disks of adjacent levels. Long term follow up studies have confirmed that fusion of the diseased cervical vertebral bodies disrupts the biomechanical balance and leads to symptomatic adjacent level degeneration [15–17].

In the 2 year follow up study of Ishihara et al., 19 patients among 112 who underwent ACDF suffered from symptomatic adjacent level degeneration. Seven of those 19 patients had to receive a second surgery alleviate the symptoms of adjacent level degeneration [18]. Different internal fixation systems have been applied to increase the room of movement at the site of surgery and adjacent levels, but the results have been mostly unsatisfactory. Cervical disk arthroplasty have provided clinicians with a novel approach to solve this problem. With the development of commercially available cervical disk prosthesis, TDR has become another option for the treatment of cervical spondylosis [19, 20].. Since the indication of ACDF and TDR are mostly the same, several RCTs were carried out comparing the efficacy of ACDF and TDR [21–24]. However, most of those studies have relatively short follow up time. Results of this 8 year follow up study indicate that TDR is superior to ACDF concerning VAS pain scores, ODI scores, ROM and SF36 scores. The reason why patient satisfaction is not significantly different between groups is possibly because most patients in both groups achieved the expected treatment results by the surgical treatment.

Retrospective nature of the current study makes it liable to patient selection bias. In our patient series, TDR is more costly than ACDF, and patients who chose to receive TDR are always economically better off than those who chose to receive ACDF. It is possible that patients who receive TDR have access to more resources to physical and mental wellbeing, this could be translated into the favorable treatment result for TDR group. However, the basic patient characteristics are similar between the two groups, and it is possible that the current study did not suffer from serious patient selection bias. More long term follow up studies, RCTs and meta-analysis can be carried out to further evaluate the safety and efficacy of those two methods.

## Conclusion

TDR is superior to ACDF concerning ODI scores, VAS pain scores, ROM and SF36. It can be applied as the main approach for patients with single level cervical disk herniation.

## Abbreviations

ACDF: Anterior cervical discectomy and fusion
ASIA: American Spinal Injury Association
JOA: Japanese Orthopedic Association scores
ODI: Oswestry Disability Index
ROM: Range of motion
TDR: Total disk replacement
VAS: Visual analog scale
